# Decaprenylphosphoryl-β-D-Ribose 2′-Epimerase, the Target of Benzothiazinones and Dinitrobenzamides, Is an Essential Enzyme in *Mycobacterium smegmatis*

**DOI:** 10.1371/journal.pone.0016869

**Published:** 2011-02-08

**Authors:** Paul K. Crellin, Rajini Brammananth, Ross L. Coppel

**Affiliations:** Department of Microbiology, Australian Research Council Centre of Excellence in Structural and Functional Microbial Genomics, Monash University, Victoria, Australia; French National Centre for Scientific Research - Université de Toulouse, France

## Abstract

**Background:**

The unique cell wall of bacteria of the suborder Corynebacterineae is essential for the growth and survival of significant human pathogens including *Mycobacterium tuberculosis* and *Mycobacterium leprae*. Drug resistance in mycobacteria is an increasingly common development, making identification of new antimicrobials a priority. Recent studies have revealed potent anti-mycobacterial compounds, the benzothiazinones and dinitrobenzamides, active against DprE1, a subunit of decaprenylphosphoribose 2′ epimerase which forms decaprenylphosphoryl arabinose, the arabinose donor for mycobacterial cell wall biosynthesis. Despite the exploitation of *Mycobacterium smegmatis* in the identification of DprE1 as the target of these new antimicrobials and its use in the exploration of mechanisms of resistance, the essentiality of DprE1 in this species has never been examined. Indeed, direct experimental evidence of the essentiality of DprE1 has not been obtained in any species of mycobacterium.

**Methodology/Principal Findings:**

In this study we constructed a conditional gene knockout strain targeting the ortholog of *dprE1* in *M. smegmatis*, *MSMEG_6382*. Disruption of the chromosomal copy of *MSMEG_6382* was only possible in the presence of a plasmid-encoded copy of *MSMEG_6382*. Curing of this “rescue” plasmid from the bacterial population resulted in a cessation of growth, demonstrating gene essentiality.

**Conclusions/Significance:**

This study provides the first direct experimental evidence for the essentiality of DprE1 in mycobacteria. The essentiality of DprE1 in *M. smegmatis*, combined with its conservation in all sequenced mycobacterial genomes, suggests that decaprenylphosphoryl arabinose synthesis is essential in all mycobacteria. Our findings indicate a lack of redundancy in decaprenylphosphoryl arabinose synthesis in *M. smegmatis*, despite the relatively large coding capacity of this species, and suggest that no alternative arabinose donors for cell wall biosynthesis exist. Overall, this study further validates DprE1 as a promising target for new anti-mycobacterial drugs.

## Introduction

**Figure 1:**
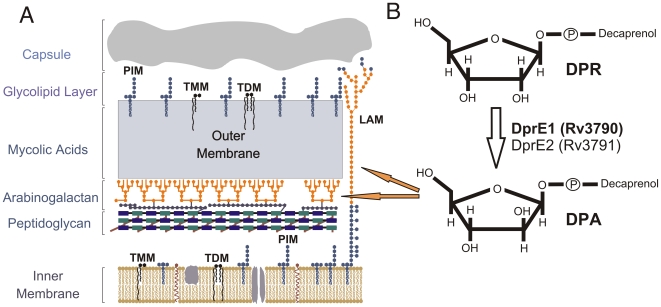
The Mycobacterial cell wall and pathway for DPA biosynthesis. **A**. The mycobacterial cell wall is a multilayered structure containing many components unique to these and closely related bacteria, including phosphatidylinositol mannosides (PIM), lipoarabinomannans (LAM), trehalose monomycolates (TMM), trehalose dimycolates (TDM) mycolic acids and arabinogalactan. The biosynthetic pathways for these unique components are a rich source of potential drug targets. Arabinose sugars are shown in orange. **B**. Decaprenylphosphoryl arabinose (DPA) is formed by the epimerization of decaprenylphosphoryl ribose (DPR) by DprE1 (Rv3790) and DprE2 (Rv3791). DPA serves as an arabinose donor in cell wall biosynthesis, contributing to arabinogalactan and LAM assembly (orange arrows).

The genus *Mycobacterium* contains several species of medical importance, most notably the re-emerging human pathogen *Mycobacterium tuberculosis* that causes nearly two million deaths each year, the most by any single infectious agent. Despite the availability of a vaccine, the number of infected individuals worldwide continues to increase, as does the prevalence of drug-resistant forms of *M. tuberculosis* [1]. A key virulence factor of Mycobacterium is the highly hydrophobic cell wall that is common to bacteria of the suborder Corynebacterineae and forms the interface between host and pathogen (Fig. 1A). The cell wall core is composed of peptidoglycan covalently linked to arabinogalactan esterified with mycolic acids, forming the mycolyl-arabinogalactan-peptidoglycan or ‘mAGP’ complex [2]. It also contains a series of free glycolipids including trehalose monomycolates (TMM), trehalose dimycolates (TDM), phosphatidylinositol mannosides (PIM) and lipoarabinomannans (LAM) that facilitate interactions with host cells [3]. The essentiality of the core for mycobacterial growth and survival means that enzymes involved in synthesis of core structures are considered excellent targets for drug development [4]. Indeed, most current front-line TB drugs target some aspect of cell wall synthesis [5].

The emergence of drug-resistant strains of *M. tuberculosis* [5], [6] is a disturbing development that highlights the need for new anti-mycobacterial compounds, particularly those with the capacity to kill persistent bacteria. Recent studies have described two classes of potent compounds with specific activities against mycobacteria. Dinitrobenzamide derivatives (DNB), originally identified in a high-throughput, fluorescence microscopy-based screen of a library of small molecules, showed potent anti-mycobacterial efficacy including activity against extensively drug resistant (XDR) *M. tuberculosis* strains, and little significant host cell toxicity [7]. Most significantly, these compounds could inhibit *M. tuberculosis* replication within macrophages. Further studies identified the mycobacterial cell wall as the target for inhibition, specifically the arabinan layers of arabinogalactan and LAM, and revealed that the arabinose donor for these layers, decaprenylphosphoryl arabinose (DPA), was not being produced from its substrate, decaprenylphosphoryl ribose (DPR, Fig. 1B). This finding strongly suggested that DNBs were targeting the heteromeric decaprenylphosphoribose 2′ epimerase encoded by the *Rv3790* (*dprE1*) and *Rv3791* (*dprE2*) genes of *M. tuberculosis* [8]. This was further supported by the finding that strains carrying a C387G mutation in DprE1 were DNB-resistant [7]. While DprE2 is also necessary for the epimerisation reaction, there is evidence of redundancy at this step [9], making DprE2 a less attractive target for drug intervention.

The potential importance of DprE1 as a drug target was further reinforced by studies on a separate set of nitro-compounds related to DNBs. Synthesis of a series of sulfur-containing heterocycles led to the identification of nitro-benzothiazinones (BTZ), a class of compounds with potent anti-mycobacterial activities [10]. These compounds were found to have very low MICs for *M. tuberculosis* and the non-pathogenic model species *Mycobacterium smegmatis*, were more potent against intracellular bacilli than front line TB drugs isoniazid and rifampin, relatively non-toxic [11] and active against multidrug-resistant clinical isolates of *M. tuberculosis* [12]. To identify the BTZ target(s), two genetic approaches were employed. The first used cosmids and subcloning to identify the DNA region responsible for BTZ resistance in *M. smegmatis* while the second involved identification and characterization of BTZ-resistant strains of *M. smegmatis*, *M. bovis* BCG, and *M. tuberculosis*. Both approaches revealed the respective DprE1 orthologs as the target. In addition, membrane preparations from wild-type *M. smegmatis* were used to show that the epimerisation reaction was inhibited by BTZ while membranes from resistant strains were resistant to such inhibition [11]. *M. smegmatis* was also exploited to identify a novel mechanism of resistance to BTZ involving overexpression of the nitroreductase NfnB [13].

Despite the exploitation of *M. smegmatis* in the identification of DprE1 as the target for BTZ and in exploring mechanisms of BTZ resistance, the essentiality of the putative DprE1 ortholog in this species has never been examined. Indeed, to our knowledge, the essentiality of DprE1 has not been experimentally investigated in any species of Mycobacterium or Corynebacterium. The *Corynebacterium glutamicum* ortholog (NCgl0187) could not be deleted by Meniche and colleagues [9] and the *M. tuberculosis* ortholog (Rv3790) is thought to be essential based on a lack of insertions in transposon mutagenesis experiments [14], however, in both cases, direct experimental evidence of essentiality is lacking. In this study we demonstrate through direct experimental approaches that MSMEG_6382 is essential for the growth of *M. smegmatis*. We show that the *MSMEG_6382* gene can only be disrupted in the presence of a second copy which, when removed, leads to a cessation of growth *in vitro*. Our findings indicate a lack of redundancy in DPA synthesis in *M. smegmatis*, despite the organism's relatively large coding capacity, and suggest that no alternative arabinose donors exist in this species. Overall, this study further validates DprE1 as a candidate for intervention by new generations of anti-mycobacterial drugs.

## Results and Discussion

### MSMEG_6382 is the ortholog of DprE1 in *M. smegmatis*

**Figure 2:**
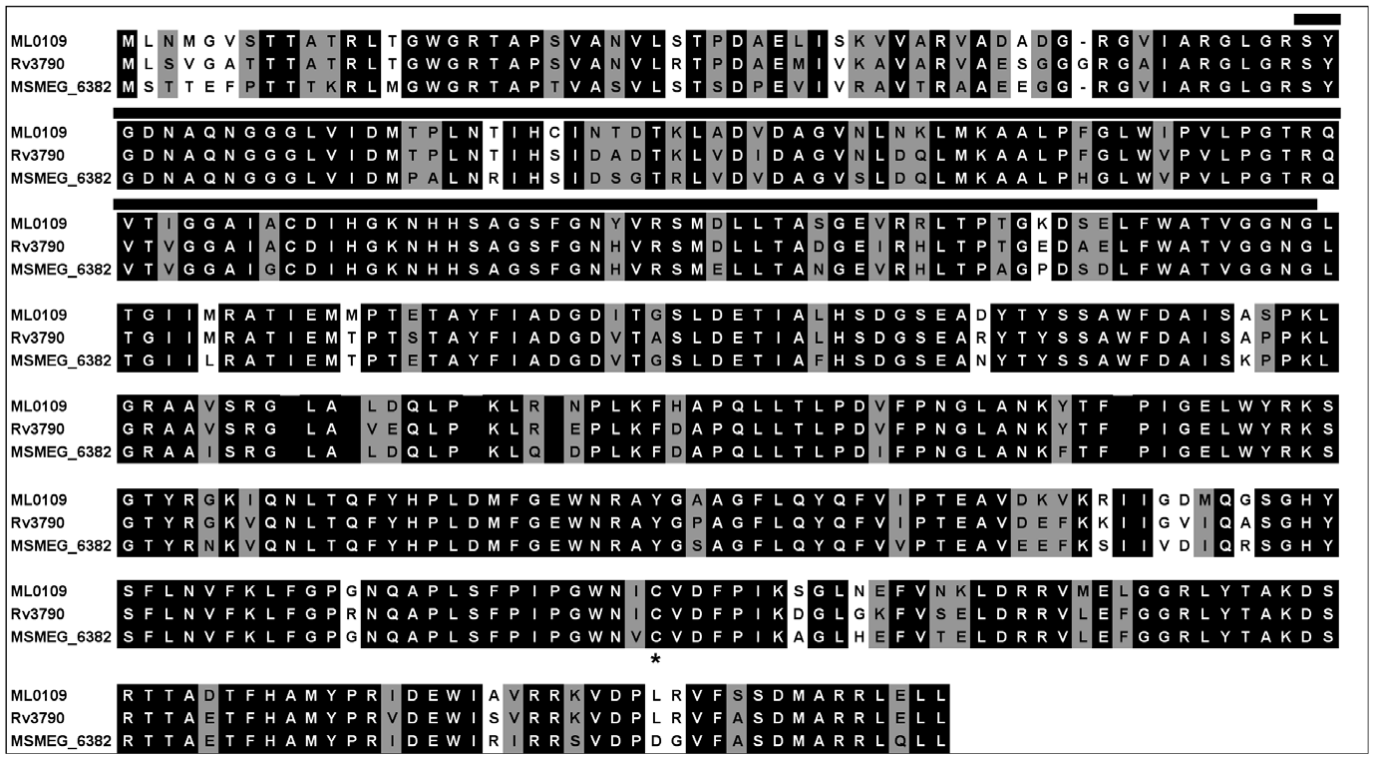
DprE1 alignment. Homologs from *M. leprae* (ML0109), *M. tuberculosis* (Rv3790) and *M. smegmatis* (MSMEG_6382) were aligned using CLUSTALW [25]. Residues that are completely conserved are reverse shaded while similar residues are indicated in grey. The putative FAD-binding domain is shown by a solid line. The conserved cysteine residue that is altered in BTZ-resistant mycobacteria [11] is indicated by an asterix.

MSMEG_6382 is 84% identical/91% similar to Rv3790 (designated DprE1 [11]) from *M. tuberculosis* and 82% identical/91% similar to ML0109 from *M. leprae* (Fig. 2). Orthologs can also be clearly identified in *Mycobacterium avium* (MAV_0232, 75%/84%), *Mycobacterium bovis* (Mb3819, 84%/91%), *Corynebacterium diphtheriae* (DIP1062, 72%/82%), *C. glutamicum* (NCgl0187, 68%/80%) *Nocardia farcinica* (nfa1970, 73%/85%) and *Rhodococcus spp.* (RHA1.ro04078, 80%/87%). The cysteine residue (Cys387 of Rv3790, Fig. 2) that results in BTZ resistance when mutated is conserved in all of these species with the exception of *M. avium* which is naturally BTZ-resistant [11].

### Construction of a conditional knockout of MSMEG_6382 (DprE1) in *M. smegmatis*

To gain insights into the importance of MSMEG_6382, we attempted to disrupt the *MSMEG_6382* gene by insertional inactivation with a drug resistance cassette. Despite several attempts, we were unable to generate this mutant, a result similar to that reported by Meniche *et al* who were unable to disrupt the *C. glutamicum* ortholog, NCgl0187 [9]. While the inability to generate a viable mutant raises the possibility that the enzyme might be essential to the organism, it is not proof of essentiality since other technical issues may be responsible. To investigate this further, we employed a genetic approach to assess the essentiality of MSMEG_6382 (see Materials and Methods for details) that had been used previously to assess the essentiality of the cell wall lipase/thioesterase, Rv3802c [15].

**Figure 3:**
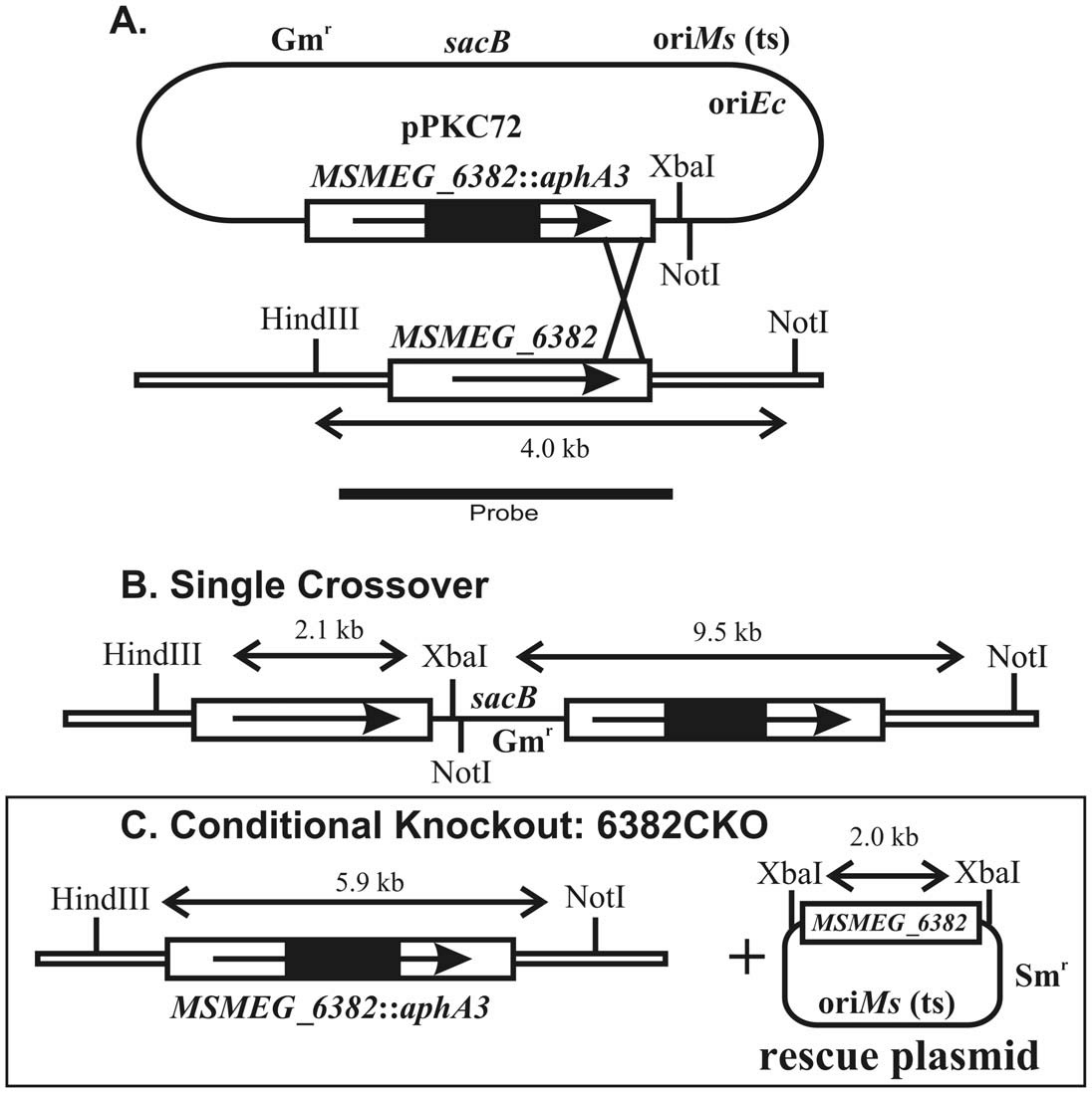
Genetic strategy for conditional disruption of *MSMEG_6382*. **A**. The recombination plasmid pPKC72 contained a cloned copy of *MSMEG_6382* interrupted by a non-polar kanamycin resistance cassette (*MSMEG_6382*::*aphA3*), a gentamycin resistance marker (Gm^r^), a temperature-sensitive replication origin for *M. smegmatis* (ori*Ms* (ts)), a replication origin for *E. coli* (ori*Ec*) and a counterselectable marker encoding sucrose sensitivity (*sacB*). The construct was introduced into *M. smegmatis* at the permissive temperature (30°C). Integration of the plasmid by a single crossover at the position indicated was detected by growing the cells at the non-permissive temperature (42°C) in the presence of kanamycin. **B**. Genetic map of the single crossover, showing key restriction sites and fragments. **C.** Culturing the single crossover strain containing a rescue plasmid encoding *MSMEG_6382* gave rise to a disrupted copy of *MSMEG_6382* in the chromosome, producing the conditional knockout (6382CKO). Since the disruption of *MSMEG_6382* coincided with the loss of the *sacB* gene, the conditional knockout strain could be selected on sucrose plates. Double lines indicate chromosomal DNA, single lines indicate plasmid DNA. Restriction fragments hybridising to the *MSMEG_6382-*specific probe are shown in kilobases (kb).

**Figure 4:**
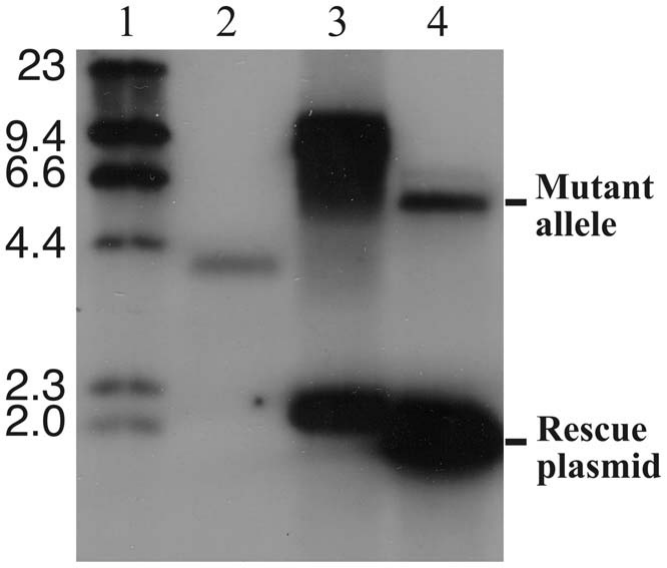
Conditional disruption of *MSMEG_6382*. Southern blot of genomic DNA digested with *Hin*dIII/*Not*I/*Xba*I. Lane 1, DNA molecular weight DNA markers of the sizes indicated (kilobases, kb); lane 2, wild-type *M. smegmatis* mc^2^155; lane 3, single crossover strain used to derive the conditional knockout; lane 4, conditional knockout of *MSMEG_6382*, designated 6382CKO.

This strategy used homologous recombination at the *MSMEG_6382* locus to disrupt the chromosomal copy of *MSMEG_6382* in the presence of a “rescue” plasmid carrying an intact copy of the same gene (Fig. 3). Briefly, the gene and flanking DNA was PCR amplified and cloned into an *Escherichia coli* vector followed by insertion of a DNA fragment carrying the *aphA3* gene, encoding kanamycin resistance, into *MSMEG_6382*. This fragment was then subcloned into pPR27, a vector with a temperature-sensitive origin of replication for *M. smegmatis*, a gentamycin resistance gene and a gene encoding sucrose sensitivity, *sacB* (Fig. 3A). This plasmid was introduced into *M. smegmatis* mc^2^155 and a single crossover strain (Fig. 3B) was selected on kanamycin plates and confirmed by Southern hybridisation (Fig. 4, lane 3). The single crossover strain carried both an intact and a disrupted copy of MSMEG_6382 (Fig. 3B).

This single crossover strain was then used to initiate a second crossover event in the presence of a rescue plasmid carrying an intact *MSMEG_6382* gene (Fig. 3C). The rescue plasmid was created by PCR cloning *MSMEG_6382* into the vector pCG76, which carries a temperature sensitive origin of replication for *M. smegmatis* and a streptomycin resistance gene [16]. Following electroporation of the rescue plasmid into the single crossover strain, allelic replacement of the chromosomal MSMEG_6382 with the disrupted copy was successfully achieved, giving rise to a “conditional knockout” strain that carried a disrupted chromosomal copy of *MSMEG_6382* and an intact copy on the rescue plasmid (Fig. 3C). The conditional knockout was confirmed by Southern hybridisation (Fig. 4, lane 4), with the rescue plasmid forming a strong band due to the presence of multiple copies. The conditional knockout strain was designated 6382CKO.

### MSMEG_6382 (DprE1) is essential for the growth of *M. smegmatis*

**Figure 5:**
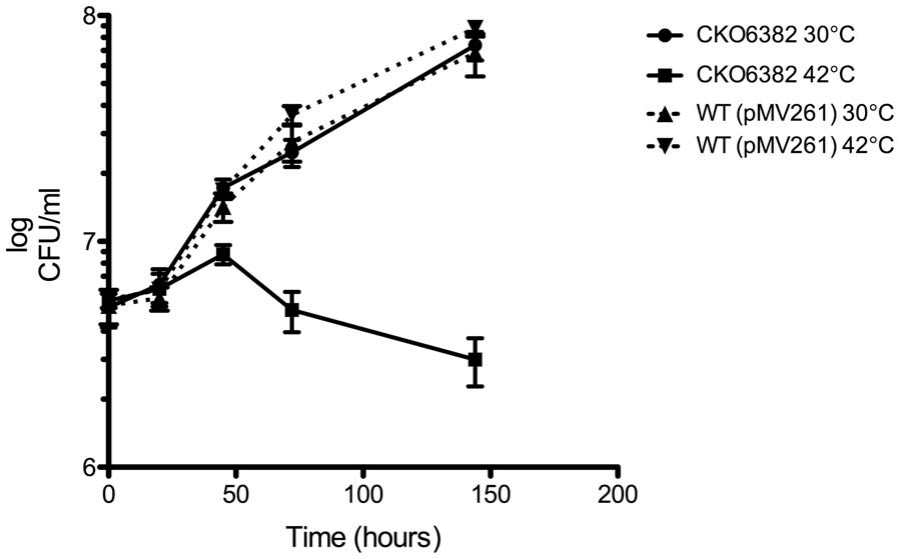
MSMEG_6382 is essential for growth of *M. smegmatis* in LB broth. 6382CKO was cultured at 30°C in LB containing Kn and Sm. At saturation, 5 ml was used to inoculate 200 ml of LB/Kn medium that had been prewarmed at the permissive (30°C •) or non-permissive (42°C ▪) temperature. Incubation was continued at the two temperatures and both cultures were sampled regularly with serial dilutions plated on LB plates containing Kn to determine colony forming units (CFUs) per ml. A wild-type *M. smegmatis* mc^2^155 strain containing the kanamycin resistance plasmid pMV261 was included as a control (30°C ▴, 42°C ▾). Unbroken lines represent the 6382CKO strain while broken lines represent the wild type (pMV261) control strain. These data represent means of triplicate samples ± standard deviation and are representative of three independent experiments.

If *MSMEG_6382* is essential, then 6382CKO should be reliant on the rescue plasmid for growth. The plasmid has a temperature-sensitive origin of replication and can replicate at 30°C but not at 42°C and is cured from the bacterial cells over several generations of growth at 42°C [16]. To determine whether is 6382CKO is reliant on the rescue plasmid, the strain was grown to saturation at 30°C then diluted into fresh LB broth pre-warmed at 30°C and 42°C. Samples were taken regularly from the two cultures over several days and serial dilutions prepared for plating on LB+Kn plates to determine the number of viable bacteria as colony forming units (cfu) per ml. The 6382CKO strain continued to grow in the 30°C culture for the duration of the experiment (Fig. 5). In contrast, growth continued at 42°C for just 2 days and then declined over the next 4 days. This “lag” is not unexpected because several generations must be completed in order for the rescue plasmid to be cured from the population. A control culture of wild-type *M. smegmatis* mc^2^155 carrying the kanamycin resistance plasmid pMV261 grew well at both temperatures, and at a similar rate to 6382CKO at 30°C, showing that the cessation of 6382CKO growth at 42°C was not simply due to the temperature change. These data show that MSMEG_6382 is essential for the growth of *M. smegmatis* in LB broth, the first time that a DprE1 ortholog has been shown to be essential in a mycobacterial species.

To determine whether MSMEG_6382 is essential for growth in other media as well, we attempted to repeat the growth curve experiment in Middlebrook 7H9 broth, a medium used for pathogenic mycobacteria but also suitable for *M. smegmatis*. Surprisingly, 6382CKO failed to grow in this medium, even at 30°C, while wild-type *M. smegmatis* grew well. Preparing a saturated pre-culture in LB broth followed by dilution into 7H9 also resulted in no growth. This result suggests that the 6382CKO strain has a moderate cell wall defect even at 30°C which renders it non-viable in particular media. Middlebrook 7H9 has a lower salt concentration than LB broth which may lead to lysis of enfeebled strains. Since 6382CKO contains multiple copies of the *MSMEG_6382* gene, we propose that overexpression of MSMEG_6382 is occurring in this strain resulting in aberrant cell wall synthesis that lead to an inability to grow in media with low osmotic pressure, such as Middlebrook 7H9.

**Figure 6:**
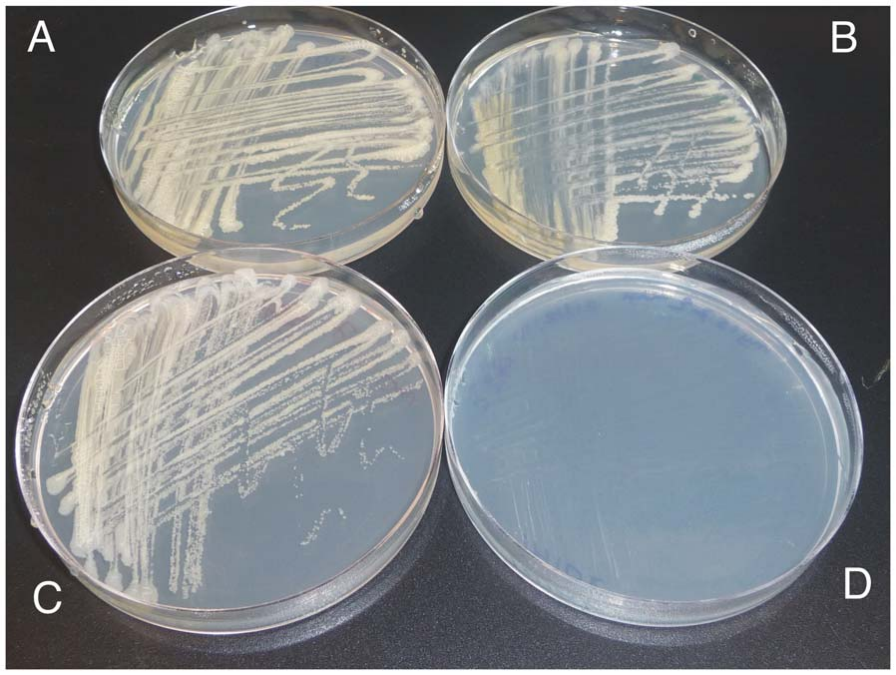
MSMEG_6382 is essential for growth of *M. smegmatis* on Middlebrook agar. The conditional knockout strain 6382CKO was cultured at 30°C on Middlebrook 7H10 agar containing Kn and Sm, then subcultured onto Middlebrook 7H10 agar containing Kn at 30°C and 42°C and examined for growth. Wild-type *M. smegmatis* mc^2^155 control strain cultured at 30°C (**A**) and 42°C (**B**) on Middlebrook 7H10 agar without antibiotics; 6382CKO strain cultured at 30°C (**C**) and 42°C (**D**) on Middlebrook 7H10 agar containing Kn.

Despite a lack of growth in liquid Middlebrook broth, we found that 6382CKO grew as well as wild-type on solid Middlebrook 7H10 agar. To investigate whether MSMEG_6382 is essential for growth on 7H10 we incubated 6382CKO at 30°C and 42°C along with a wild-type control strain. As shown in Figure 6, the control strain grew at both temperatures while 6382CKO failed to grow at 42°C. Some growth was visible in the primary streak (Fig. 6D) but this was expected because several generations of growth at 42°C are required to cure the rescue plasmid, as described above. Overall, we were able to conclude that the *MSMEG_6382* gene is essential for *M. smegmatis* growth under at least two different culture conditions.

To determine the structural consequences of a loss of MSMEG_6382 on *M. smegmatis*, we examined the 6382CKO strain, cultured at 42°C for 5 days on LB agar, by scanning and transmission electron microscopy. Surprisingly, the cells were found to be intact (data not shown), suggesting a bacteriostatic rather than bacteriolytic effect. This is in contrast to a conditional knockout of another cell wall biosynthesis enzyme, Rv3802c, which had a dramatic loss of cellular integrity at 42°C [15]. It is also inconsistent with the finding that exposure of *M. smegmatis* and *M. tuberculosis* to one BTZ derivative, BTZ043, resulted in a swelling of the poles of the cells followed by lysis [11]. If BTZ043 kills *M. smegmatis* by inhibiting MSMEG_6382, we would predict that drug treated cells and our conditional knockout cured of the rescue plasmid would look the same. However, the experimental conditions of the two studies are significantly different and Makarov et al used extremely high concentrations of the drug to induce pole swelling and lysis. Also, although the reasons for the discrepancy are unclear, it is possible that extending our growth curve beyond 5 days might reveal cell lysis. Unfortunately, the strong selective pressure leads to the appearance of revertents after 5 days at 42°C that overgrow the culture, so we were unable to extend the experiment beyond a 5 day period. We have isolated such revertents from CKO6382 and they fail to grow on Sm at 30°C or 42°C, suggesting loss of the rescue plasmid. Revertents are also readily isolated from other conditional knockouts we have constructed, including a published strain [15]. The most likely explanation is that the intact copy of the gene on the rescue plasmid recombines into the genome under strong selective pressure to restore viability at 42°C.

### No redundancy in the DprE1-catalyzed reaction in *M. smegmatis*

*M. smegmatis* is often used as a model for pathogenic mycobacteria due to its relatively fast growth rate and well-defined genetic systems. As a saprophytic bacterium with the capacity to adapt to a range of conditions, it possesses a large genome of 7.0 Mb encoding 6,829 genes. By comparison, the *M. tuberculosis* H37Rv genome is 4.4 Mb (3,918 genes) [17] while the genome of the obligate pathogen *Mycobacterium leprae* comprises just 3.3 Mb (2,720 genes) [18]. The relatively large codon capacity of *M. smegmatis* increases the potential for redundancy in cell wall biosynthetic (and other) pathways. However, our findings suggest that there is no redundancy in the DprE1 step in *M. smegmatis*. Based on this finding, we predict that the epimerisation reaction will be essential in all mycobacteria and corynebacteria, making DprE1 inhibitors effective against a range of problematic pathogens. The second component of the epimerisation complex, DrpE2, shows less potential as a drug target because disruption of the *C. glutamicum* ortholog is possible due to compensation by a functionally equivalent enzyme. Enzymes that could compensate for the DprE2 of *M. tuberculosis* (Rv3791) have also been described [9], although Rv3791 has been predicted to be essential in *M. tuberculosis* [14]. In addition, we have successfully disrupted the *dprE2* ortholog of *M. smegmatis* (data not shown), suggesting redundancy in this organism as well. Studies on this *dprE2* (*MSMEG_6385*) mutant are currently in progress.

### No alternative arabinose donors for cell wall biosynthesis in *M. smegmatis*

The essentiality of DprE1 in *M. smegmatis* also suggests that there are no alternative arabinose donors in this species that can compensate for a lack of DPA. If present, such donors would be expected to restore viability to a *dprE1* mutant. A possible alternative arabinose donor has been described in *M. smegmatis* (Wolucka, 2008). This compound, which contains an activated D-arabinose that could theoretically serve as a donor, was identified as a partially saturated β-D-arabinosyl-1-monophospho-octahydroheptaprenol (Wolucka and Hoffmann, 1994). It is possible that this species could be also formed by the DprE1/DprE2 epimerization complex but ribosylated derivatives of C_35_-octahydroheptaprenyl phosphate have never been described (Wolucka, 2008), making this an unlikely possibility. The biosynthetic pathway and biological role(s) of this molecule remain unknown.

### DprE1 as a “magic” mycobacterial drug target

In this study we have employed a genetic approach to provide further evidence of the essentiality of DprE1 in mycobacteria. Although two new classes of antibiotics highly specific for mycobacteria seem to target this enzyme [7], [11], and indirect evidence of essentiality had been reported in *C. glutamicum* and *M. tuberculosis* [9], [14], direct experimental evidence of essentiality has never been presented before. The essentiality of DprE1 in *M. smegmatis*, combined with its conservation in all sequenced mycobacterial genomes, suggests that DPA synthesis is essential in all mycobacteria and not subject to redundancy. DprE1 has been described as a “magic” drug target [19] and our findings are in full agreement with this assessment.

## Materials and Methods

### Growth and manipulation of *E. coli* and *M. smegmatis*

Bacteria were routinely cultured at 30°C, 37°C or 42°C in Luria Burtani (LB) medium or Middlebrook medium (7H9 broth or 7H10 agar, Difco) supplemented with kanamycin (Kn, 20 µg/ml), streptomycin (Sm, 20 µg/ml), ampicillin (Ap, 100 µg/ml) and sucrose (10% w/v), as appropriate. Tween-80 was added to 0.05% (v/v) to reduce clumping in mycobacterial liquid cultures. Competent *M. smegmatis* cells were prepared as described [20] and electroporated using a Biorad Gene Pulser with the following settings: 2.5 kV, 1000Ω, 25 µF.

### DNA Manipulations

Polymerase chain reactions were performed using Proofstart DNA Polymerase (Qiagen) according to the manufacturer's instructions. Reactions consisted of a hot-start (95°C, 5 min) followed by 35 cycles of denaturation (95°C, 1 min), annealing (55°C, 1 min) and extension (72°C, 2 min). Restriction enzymes and T4 DNA polymerase were from Roche or New England Biolabs. Genomic DNA was prepared from mycobacteria as described [21]. Southern blots [22] were performed using digoxygenin-labelled probes (Roche) according to manufacturer's instructions.

### Construction and analysis of a conditional knockout of MSMEG_6382

Rv3790 and flanking DNA was PCR amplified from *M. smegmatis* mc^2^155 genomic DNA as a 2 kb fragment using primers A (5′- GATCAAGCTTACGCCCTCGATCGTCCTG-3′) and B (5′- TGCAGCGGCCGCCAGCGCGTGCAGCGAG -3′), digested at the underlined restriction sites for *Hin*dIII and *Not*I, and cloned into *Hin*dIII-*Not*I-digested pUC18 [23]. A 1.9 kb non-polar kanamycin resistance cassette carrying the *aphA3* gene was then inserted at a unique *Eco*RV site within MSMEG_6382. Finally, the 3.9 kb *Hin*dIII-*Not*I fragment containing *MSMEG_6382*::*aphA3* was transferred to *Bam*HI-digested pPR27 [24], following T4 polymerase treatment of both insert and vector, creating the final recombination plasmid, pPKC72.

To generate single crossovers, pPKC72 was introduced into *M. smegmatis* mc^2^155 by electroporation and selecting kanamycin resistant clones at 30°C. A 10 ml LB broth containing kanamycin was inoculated with culture single colony and grown for five days at 30°C to saturation. Serial dilutions were plated onto LB+Kn plates at 42°C and incubated for four days to select for potential single crossovers. Colonies were screened for incorporation of pPKC72 into the chromosome by growing 10 ml LB/Kn cultures to saturation at 42°C, extracting genomic DNA, digesting with HindIII/NotI/XbaI and performing a Southern hybridization with a probe specific for *MSMEG_6382*. A single crossover, designated Myc51, was selected for further analysis.

To derive a double crossover (a conditional knockout) a rescue plasmid containing *MSMEG_6382* carried on a 2.0 kb XbaI fragment cloned into the temperature-sensitive plasmid pCG76 [16] was introduced into Myc51 by electroporation. The XbaI fragment did not contain sequences upstream of *MSMEG_6382* so gene expression is reliant on vector promoters. Transformants were selected on LB+Kn+Sm plates at 30°C and a single colony was grown to saturation LB+Kn+Sm broth at 30°C. Serial dilutions were plated on LB+Kn+Sm plates containing sucrose and incubated at 30°C. Potential conditional knockout clones were grown in 10 ml LB/Kan/Sm to saturation followed by genomic DNA extraction, digestion with HindIII/NotI/XbaI and Southern blotting using a MSMEG_6382-specific probe. A confirmed conditional knockout strain was designated 6382CKO.

To determine growth curves, 6382CKO and control strains were cultured in 10 ml LB/kan at 30°C for 3 days, then 5 ml was added to 200 ml of LB/Kan that had been pre-warmed to 30°C or 42°C. The cultures were sampled daily and serial dilutions plated onto LB/Kan at 30°C. Following 5 days incubation, colonies were counted to determine the number of colony forming units per ml.
